# Home-Based, Low-Intensity, Gamification-Based, Interactive Physical-Cognitive Training for Older Adults Using the ADDIE Model: Design, Development, and Evaluation of User Experience

**DOI:** 10.2196/59141

**Published:** 2024-10-29

**Authors:** Teerawat Kamnardsiri, Sirintip Kumfu, Peeraya Munkhetvit, Sirinun Boripuntakul, Somporn Sungkarat

**Affiliations:** 1 Department of Digital Game, College of Arts, Media, and Technology, Chiang Mai University, Chiang Mai, Thailand; 2 Department of Physical Therapy, Faculty of Associated Medical Sciences, Chiang Mai University, 110 Intawaroros Rd, Sripoom, Chiang Mai, 50200, Thailand, 66 53949249; 3 Department of Occupational Therapy, Faculty of Associated Medical Sciences, Chiang Mai University, Chiang Mai, Thailand

**Keywords:** exergame, physical-cognitive training, computer-based interventions, gamification, older adults, instructional design model, low-intensity

## Abstract

**Background:**

Declines in physical and cognitive function are natural biological processes, leading to an increased risk of falls. Promising evidence suggests that combined physical-cognitive exercise has beneficial effects in improving both physical and cognitive health. Although moderate-to-high exercise intensity is commonly recommended, it might be impractical for older adults facing physical limitations or contraindications. Thus, low-intensity exercise is a viable option. The main barriers to engaging in exercise in older adults include transportation, time, motivation, and enjoyment. To overcome these challenges, a home-based, gamification-based training system may provide an effective approach to enhance exercise adherence.

**Objective:**

This study aimed to develop and evaluate the usability of a low-intensity, gamification-based, interactive physical-cognitive exercise for older adults in a home-based setting.

**Methods:**

The prototype of a game-based physical-cognitive exercise was created following the ADDIE model (analysis, design, development, implementation, and evaluation) and assessed for user experience in older adults. A total of 15 older adults engaged in the game-based physical-cognitive exercise at home for 60 minutes per day, 3 days per week, for 4 weeks. The usability of the game-based training system was evaluated using the system usability scale (SUS) after completion of a 4-week training program. As for satisfaction, the 8-item Physical Activity Enjoyment Scale (PACES) questionnaire was used to assess participants’ enjoyment level after 1 week and 4 weeks of training. Descriptive statistics were used to illustrate the SUS score. A Wilcoxon signed-rank test was used to compare the PACES scores between the first week and the end of the 4-week period, with significance set at *P*<.05.

**Results:**

As for experts’ consensus, the game-based training consisted of 3 games: Ocean Diver, Road Runner, and Moving and Memorizing. The games had 3 levels of difficulty: beginner, intermediate, and advanced. A computer vision–based system was selected as the delivery platform for a home setting. The total SUS score for all participants was mean 87.22 (SD 5.76), indicating the user’s perception of the usability of a system ranging from good to excellent. At the end of the 4-week training, the total PACES score was significantly greater than the first week, suggesting an improvement in enjoyment (first week: mean 44.93, SD 3.99 vs fourth week: mean 50.53, SD 4.70; *P*=.001).

**Conclusions:**

The prototype of low-intensity, gamification-based, interactive physical-cognitive training was designed and developed using the ADDIE model, which included both experts and end users in the process. The findings showed that the exergame prototype was a usable and practical approach for a home-based setting, enhancing older adults’ enjoyment and motivation. Further research is warranted to determine the effectiveness of such gamification-based training in promoting physical and cognitive functions.

## Introduction

Aging is a natural biological process leading to a gradual decline in both physical and cognitive abilities. Physical decline in older adults, which contributes to their susceptibility to falls, includes intraindividual factors such as muscle weakness, delayed reaction time, impaired vision and proprioception, and impaired balance [1]. In addition to a decline in physical function, older adults may also experience problems in cognitive function, primarily involving executive function, attention, and processing speed, which are significant risk factors for falls [2]. Given that physical and cognitive impairments coexist in aging and are indicators of the risk of falls, it is essential to identify effective strategies to enhance both physical and cognitive functions for fall prevention.

It is well established that exercise serves as an effective strategy for fall prevention among older adults by ameliorating physiological fall risk factors such as muscle weakness and poor balance [3,4]. Accumulating evidence has demonstrated that exercise benefits not only physical function but also cognitive function. Given that cognitive impairment is an independent risk factor for falls, improving cognitive function through exercise may further contribute to fall risk reduction [5-7]. Among various types of exercise, combined physical-cognitive training (simultaneous training) has a profound effect on cognitive and physical health, reducing the risk of falls in older adults, and it is superior to single-component training [8,9]. Although moderate-to-high intensity exercise is widely recommended for reducing the risk of falls, its applicability may be limited for certain older adults with physical restrictions or contraindications. Currently, a growing body of evidence has suggested that low-intensity exercise has a positive effect on cognitive and physical performance in adults and older adults with and without pathologic conditions [10-13]. With this, a low-intensity exercise may be a promising alternative approach for fall prevention in older adults who experience physical limitations that prevent them from engaging in moderate-to-high intensity exercise.

With current technological advancements, gamification, which involves using game-based mechanics to motivate individual action and learning for a specific target, is increasingly used in health care services, especially for promoting exercise [14,15]. Among gamification-based exercises, combined physical-cognitive training has emerged through exergames that require individuals to move their bodies to interact with the game for achieving training purposes [16-18]. Previous studies have shown that exergame-based interventions improve both physical aspects (eg, balance, gait, and physical fitness) and cognitive domains (eg, executive functions, memory, and processing speed) in older adults while also facilitating enjoyment and motivation, which are critical mediators of training adherence and goal-directed achievement [19-22]. Nowadays, many commercial interactive game-based training products have emerged, such as Kinect Xbox, Nintendo Wii, Sony PlayStation, virtual reality systems, and computer vision–based applications. Amid the exergames in the commercial market, human pose estimation, which is one of the computer vision–based platforms, has rapidly developed in recent years. Specifically, human pose estimation involves extracting an individual’s joint positions from an image or video to create a skeletal shape. With this, an algorithm of machine learning creates a human pose estimation model based on dataset samples of human movements in space, which ultimately enables machines to correctly interact with individuals [23,24]. One interesting feature of computer vision–based systems using human pose estimation is that they are markerless full-body trackers that enable users to naturally interact with games in real time. Additionally, it is reliable for representing a person’s movements, resulting in proper feedback and guidance to the users during exercise [25]. Its advantages include its low cost, user-friendliness, simpler operation, and the requirement of only a personal computer, thereby increasing accessibility. Therefore, a computer vision–based system appears to be a viable alternative to delivering home-based exercise programs for older adults.

The ADDIE model is an instructional systems design framework used to design and develop learning experiences that involve identifying the requirements and understanding the solutions that learners should achieve [26]. The ADDIE model comprises 5 phases: analysis, design, development, implementation, and evaluation [27]. There has been growing interest in using the ADDIE model to develop health services for various purposes [28-32]. However, previous works utilizing the ADDIE model to develop exergames aimed at enhancing physical and cognitive functions are scarce. Considering the usability challenges faced by older adults, developers should design exergame interfaces that are user-friendly for this population. Aside from the technologies used, commercial exergames available in the market may not be specific for older people in terms of training purposes, complexity, intensity, and safety. Thus, the ADDIE model may be a useful approach for designing and developing exergames for older adults, particularly in a home environment.

Collectively, a game-based, combined physical-cognitive training that is accessible and user-friendly, boosts motivation, and is tailored to suit older adults’ capabilities would represent an efficient approach in terms of time and resources. Therefore, this study focused on developing the prototype of a home-based, low-intensity, gamification-based, interactive physical-cognitive training for older adults using the ADDIE model. Notably, the usability of the system and participants’ satisfaction will be investigated to facilitate practical implementation for community-dwelling older adults.

## Methods

### Study Design

#### Overview

**Figure 1.**
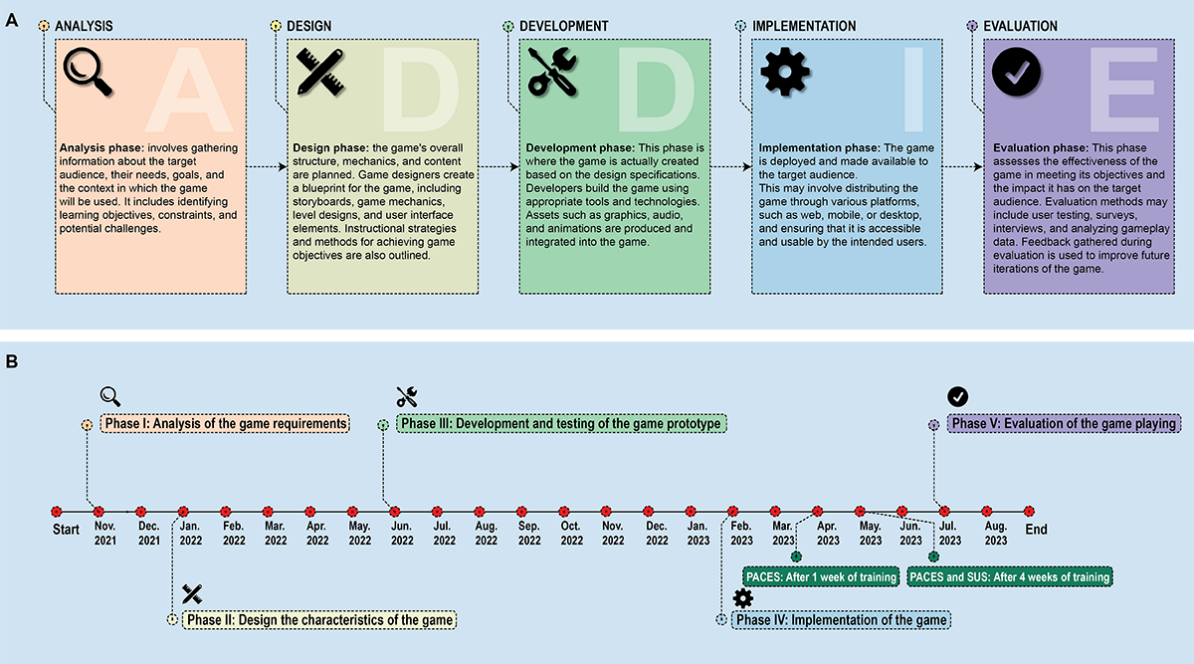
(**A**) Development process of the game-based training system using the ADDIE (analysis, design, development, implementation, and evaluation) model. (**B**) Development milestone of the game-based training system prototype.

This study utilized the ADDIE model to design and develop a low-intensity, combined physical-cognitive exercise in the form of an exergame delivered in a home-based format for older adults. The prototype of a gamification-based, interactive physical-cognitive training was built upon five phases: (1) analysis, (2) design, (3) development, (4) implementation, and (5) evaluation of target users’ feedback, response, and satisfaction. The ADDIE model is depicted in Figure 1A, and the development milestones of this study are presented in Figure 1B.

#### Phase I: Analysis Phase

During the analysis phase, a focus group expert interview was conducted to extract essential contents in terms of knowledge related to effective physical and cognitive training programs for older adults and potential core game ideas. A total of 7 experts participated in the brainstorming session, including 4 physical therapists and 1 occupational therapist (ages ranging from 38 to 60 years, with 10-25 years of experience in physical and cognitive rehabilitation for older adults, both with and without cognitive impairment), 1 game programmer (29 years old, with 4 years of experience in developing codebases for video games or related software), and 1 game artist (29 years old, with 5 years of experience in developing game mechanics and interfaces).

In this study, the core elements of the exergame consisted of two training components that were trained simultaneously: (1) a physical component at a low intensity level focusing on enhancing dynamic balance, coordination, and muscle strength and endurance of upper and lower limbs, and (2) a cognitive component focusing on memory, attention, and executive function (eg, anticipation, planning, switching, and inhibition) that closely related to balance and falls in older adults [2,33]. Regarding the delivery platform, a computer vision–based system was selected due to its low cost, accessibility, and simple operation, allowing older adults to independently administer self-training at home.

#### Phase II: Design Phase

In the design phase, the overall game structure and characteristics, including the game’s goals, mechanics, and graphical user interface elements, were integrated and fine-tuned from a practical standpoint to form a design of the game-based training system prototype. Moreover, the progression of game difficulty was divided into 3 difficulty levels (ie, beginner, intermediate, and advanced) to ensure that the difficulty of the games was appropriate for each user. After that, the game blueprint underwent a critical review (using the assessment form found in Multimedia Appendix 1) by 5 experts in the field of cognitive and physical interventions for older adults, including 2 physical therapists, 1 occupational therapist, 1 neurologist, and 1 geriatrician, with ages ranging from 40 to 56 years and experience ranging from 18 to 34 years. The content validity of the exergames was analyzed using the index of item-objective congruence (IOC). The cutoff value is a flexible criterion, with a generally accepted minimum typically being 0.75 [34].

#### Phase III: Development Phase

**Figure 2.**
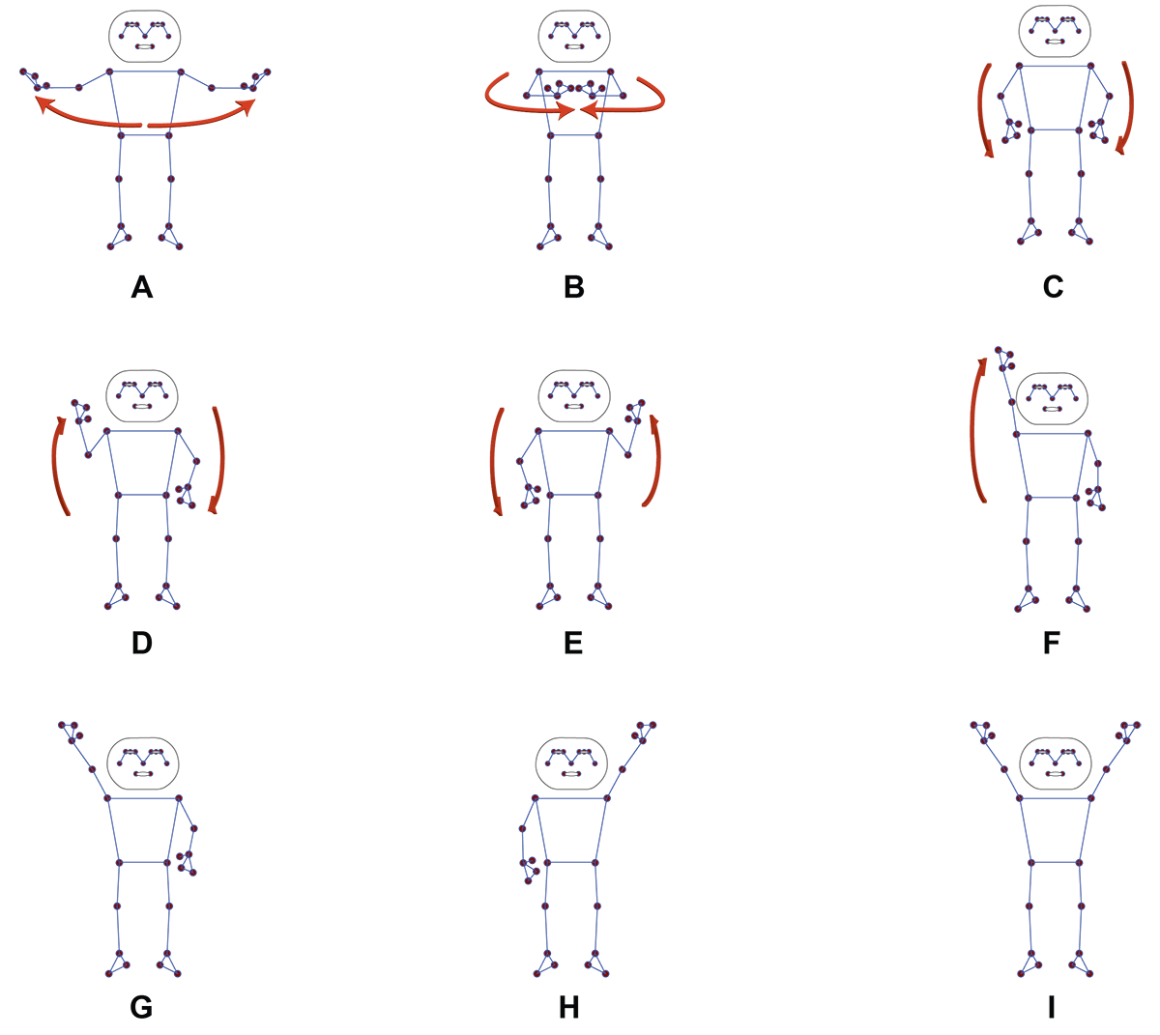
Condition of each landmark for control of the avatar in the game. (A) and (B) Moving bilateral arms horizontally to swim in a frog stroke style. (C) Dropping bilateral arms to rest. (D) and (E) Moving bilateral arms as if running to control the forward movement. (F) Raising the left or right hand over the head to collect objects. (G) Raising the right hand over the head to select choice A. (H) Raising the left hand over the head to select choice B. (I) Raising both hands over the head to select choice C.

Consensus on the game idea and design, along with comments and feedback from the experts during the design phase, was used to refine the game-based exercise prototype in the development phase. This prototype was created using the Unity 3D game engine software, incorporating computer vision techniques from BlazePose for 2D/3D pose estimation [35]. The landmarks of the BlazePose 2D/3D pose estimation, which include 33 key points, are provided in Multimedia Appendix 2. In this study, only 12 key points (of 33 points) were utilized to control the mechanics of the games. These key points included the following: (1) left_shoulder, (2) right_shoulder, (3) left_wrist, (4) right_wrist, (5) left_pinky, (6) right_pinky, (7) left_index, (8) right_index, (9) left_thumb, (10) right_thumb, (11) left_hip, and (12) right_hip. The various conditions for controlling the game’s avatar are displayed in Figure 2.

**Figure 3.**
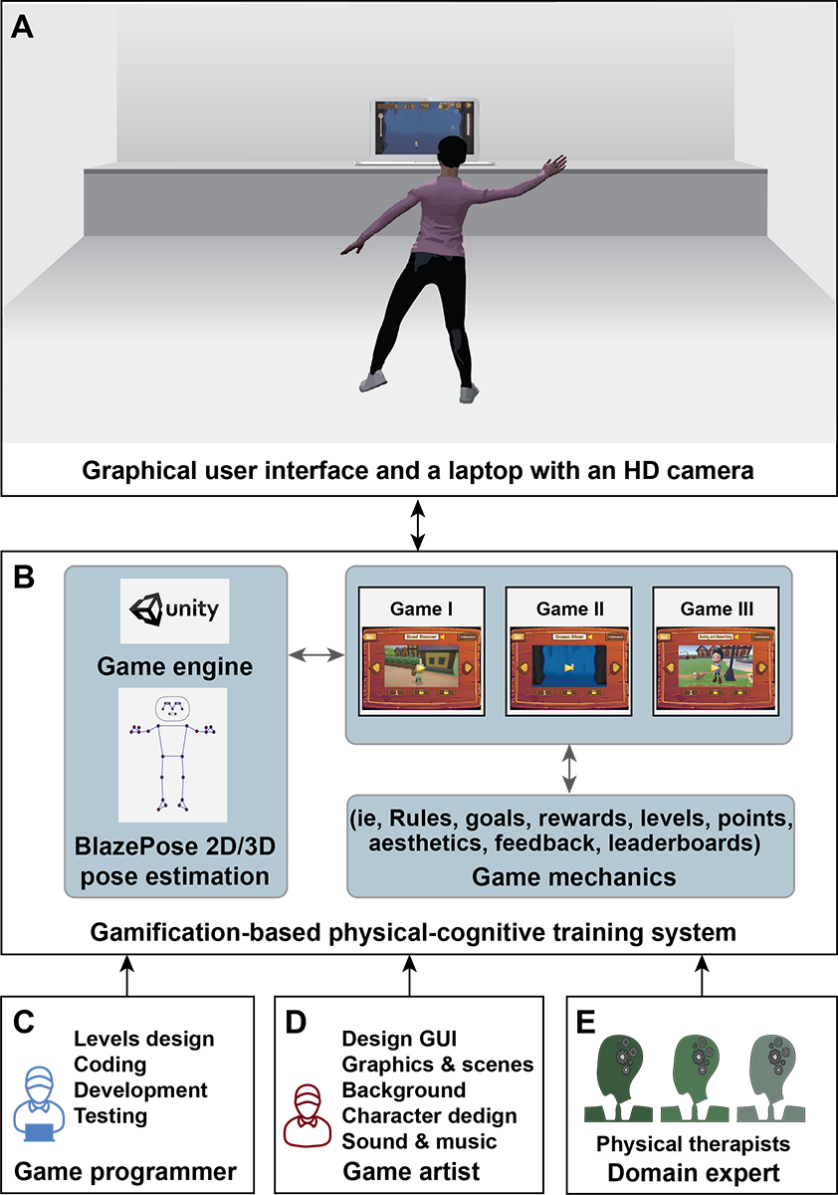
Gamification-based, interactive physical-cognitive training framework: (**A**) user interface and laptop with a camera, (**B**) system of gamification-based physical-cognitive training, (**C**) game programmer, (**D**) game artist, and (**E**) domain experts.

After completing phases I-III, the overall framework of the gamification-based, interactive physical-cognitive training system prototype, consisting of 5 components, was established (Figure 3):

1. User and the notebook with high-definition (HD) webcam sensor: The full-body movements were tracked in 3D coordinates (ie, the x-, y-, and z-axes) using 12 key points (Figure 2). The HD webcam sensor was used to track the full-body movements of the participants while they played the exergame (Figure 3A).
2. The gamification-based physical-cognitive training system: The digital game system consisted of 3 exergames (Game I: Ocean Diver; Game II: Road Runner; and Game III: Moving and Memorizing) with 3 levels of difficulty (beginner, intermediate, and advanced) and feedback (score, time, and error). Moreover, rules, goals, rewards, points, aesthetics, leaderboard, and game mechanics were used as elements of the game. The game was developed using the Unity game engine (Figure 3B).
3. Game programmer: The specialist who created the game using computer programming languages and game engine software, including level design, coding, game development, and game testing (Figure 3C).
4. Game artist: The specialist who designed and created graphical content, including the graphical user interface, character, scenes, background, sound, and music (Figure 3D).
5. Domain knowledge: The experts with core knowledge in physical and cognitive training programs for older adults, both those with and without cognitive impairment (Figure 3E).

#### Phase IV: Implementation Phase

##### Overview

In this phase, the prototype of a low-intensity, gamification-based, interactive physical-cognitive exercise was implemented to representative target users to evaluate its usability and ability to promote enjoyment. The prototype of the exergame was delivered via the Windows platform and devices, including notebooks and personal computers.

##### Recruitment and Participants

A total of 15 community-dwelling older adults were recruited from community groups such as local senior schools and senior clubs, as well as through social media advertisements. Inclusion criteria were as follows: (1) age 60 years or older, (2) ability to comprehend instructions and willingness to participate, (3) adequate vision and hearing, or correction for impairments, and (4) sufficient physical performance, indicated by scoring at least 9/12 on the Short Physical Performance Battery, to ensure safety during exercise, particularly to prevent loss of balance and falls (Multimedia Appendix 3) [36]. Exclusion criteria were as follows: (1) being diagnosed with other neuromuscular conditions that affect cognitive function and physical ability (eg, stroke, Parkinson disease, dementia, and multiple sclerosis), and (2) having major health conditions that could not be controlled (eg, acute joint pain, asthma, hypertension, diabetes mellitus, and coronary artery disease).

##### Procedures

###### System Setup and Calibration

Prior to participants beginning the interactive exergame training at their homes, the researchers made a home visit for the system’s setup, calibration, and operation. The capture volume was configured using a notebook or personal computer equipped with an HD webcam. The HD webcam was positioned on a table approximately 0.8 meters above the floor and about 2.5 meters away from the participant. The capture volume from left to right was set at approximately 3.0 meters, with the center being 1.5 meters from each side. The game was displayed on either notebook screens or computer monitors. The configuration of hardware for playing the game is provided in Multimedia Appendix 4. The game-based exercise was connected to the HD webcam sensor in the notebook or personal computer. During the calibration process, participants were instructed to move the avatar within the capture volume, with the boundaries marked by markers on the floor. Once the system had been fully set up, participants were able to use it independently.

###### User Experience Testing

The demographic information of eligible participants—including age, gender, BMI, and education level—was documented. The game goals, rules, mechanics, and controls were explained to the participants and they were provided with a demonstration. The participants interacted with the virtual game using their body and hand movements following each game’s rules (ie, Ocean Diver, Road Runner, and Moving and Memorizing). The training duration was 35 minutes, which included warm-up and cool-down periods, with a rest interval of approximately 5 minutes, resulting in a total session time of around 60 minutes. Participants were allowed to progress to the next level if they achieved a score exceeding 50% in the current level. To assess the usability and enjoyment of the prototype, participants engaged in game-based training at their homes 3 times per week for 4 consecutive weeks (a total of 12 sessions).

### Phase V: Evaluation Phase

Participants were asked to provide ratings for both the system’s usability and their enjoyment during engagement with the game-based prototype after completing the 4-week testing period. The usability of the game-based physical-cognitive training system was assessed using the system usability scale (SUS) at the end of the training period [37]. The SUS rating scale was then converted from the original score (between 0 and 60) to an SUS score ranging from 0 to 100, where an SUS score above 68 is considered an above average (“good”) or marginal acceptance level, and scores above 85 are considered an “excellent” acceptance level [38]. Additionally, the level of enjoyment during exercise engagement was assessed after the first and fourth week of training using the 8-item Physical Activity Enjoyment Scale (PACES) questionnaire, a 7-point Likert scale ranging from 1 (strongly disagree) to 7 (strongly agree) [38]. The sum of PACES scores was calculated, with a higher score indicating a greater level of enjoyment during exercise engagement. Moreover, participants were interviewed about their perceptions of various aspects of the exergame during gameplay using probing questions (Multimedia Appendix 5). In the interview, assessors asked core questions sequentially and documented the responses. Upon completing the questioning, assessors summarized the key insights from the interview to ensure a comprehensive understanding of the participant’s responses. Afterward, assessors extracted key themes from the responses provided by all participants to gather both positive and negative feedback, as well as information for improvements and refinements.

### Ethical Considerations

The research protocol received approval from the Human Ethical Review Board of the Chiang Mai University (approval number AMSEC-66EX-036). All participants were informed about the study’s purpose and procedure before providing written informed consent to enroll in the study. All data used for this study were anonymized. No compensation was provided to the participants.

### Statistical Analysis

The content validity of the exergames, as assessed by experts, was determined using the IOC, with an IOC value greater than 0.75 indicating an acceptable level of validity. Descriptive statistics were used to illustrate the demographic profile of the participants and the SUS score after 4 weeks. The Wilcoxon signed-rank test was used to address differences in the PACES scores between the first week and the end of the 4-week training period. The significance level was set at *P*<.05. Analysis was conducted using SPSS (version 21.0; IBM Corp).

## Results

### Phase I-III: Analysis, Design, and Development Phases

The game-based training system underwent a critical review by 5 experts in the field of cognitive and physical interventions for older adults. The results indicated that the content validity was highly acceptable, with IOC values ranging from 0.80 to 1.00. The summary of the consensus features of the low-intensity, gamification-based, interactive physical-cognitive exercise is presented in Table 1 and Textbox 1. This system consisted of 3 exergames (Ocean Diver, Road Runner, and Moving and Memorizing), all aimed at enhancing simultaneous physical and cognitive function in older adults. Each exergame had 3 levels of difficulty (beginner, intermediate, and advanced), in which game complexity progressed by increasing the complexity of physical and cognitive demands. The total game play time was 35 minutes. In accordance with exercise principles, warm-up and cool-down sessions were included as part of the training. The exergame prototype was delivered via a computer vision–based system that participants could operate independently in a home-based setting.

**Table 1.** Summary of the features of the developed gamification-based, interactive physical-cognitive training.

	Game I: Ocean Diver	Game II: Road Runner	Game III: Moving and Memorizing
Actions	Swimming in a frog stroke style underwater to collect predetermined objects in the correct order and collect coins as a bonus	Running along the road to collect as many predetermined objects as possible and collect coins as a bonus	Walking along the road to collect coins while listening to a story, then answering questions related to the story at the end of the game
Rules and game mechanics	Moving bilateral arms in a frog stroke style to control the avatar’s upward or downward direction Stepping to the left or right to control the avatar’s left or right movement direction	Moving bilateral arms as if running to control the avatar’s forward movement Stepping to the left or right to control the avatar’s left or right movement direction Raising the left or right arm to control the avatar to collect predetermined objects	Stepping to the left or right to control the avatar’s left or right movement direction Listening to a story and collecting coins as a bonus Answering questions related to the story at the end of the game
Virtual environment	An underwater world with aquatic animals and treasures	A suburban village with fruits, vegetables, and animals	A suburban village with coins
Goal of the game: physical components	Improve dynamic balance and coordination of upper and lower limbs Improve upper and lower limb muscle strength and endurance	Improve dynamic balance and coordination of upper and lower limbs Improve reaction and response time Improve upper and lower limb muscle strength and endurance	Improve dynamic balance Improve lower limb muscle strength and endurance
Goal of the game: cognitive components	Improve memory, attention, and executive function (ie, planning, sequencing, and inhibiting)	Improve memory, attention, and visuospatial ability	Improve memory (delayed recall) and attention

Textbox 1. **Framework of the gamification-based physical-cognitive training.**

- Physical component training progression: the difficulty levels progressed by increasing the subject’s movement speed of upper and lower limbs (eg, adding the number of objects).
- Cognitive component training progression: the difficulty levels progressed by increasing the subject’s cognitive demands to play the game (eg, adding memory and attention requirements, increasing the speed of processing, and adding the number of objects).
- Rewards: points and coins
- Estimated playtime: 35 minutes
- Health topic: gamification-based physical-cognitive training prototype
- Targeted age group: older adults (age ≥60 years)
- Short description of the gamification-based training: gamification-based physical-cognitive training is an interactive game-based training system for older adults using high-definition webcam sensor technology. It comprises 3 games involving physical training (ie, dynamic balance; coordination; reaction and response time; and upper and lower limb muscle strength and endurance) and cognitive training (ie, memory or delayed recall, attention, and executive function including planning, sequencing, inhibiting, visuospatial ability.
- End user or target player: older adults (individual self-training)
- Clinical support needed: physical therapists and related geriatrics health care professionals
- Data shared with clinician: data are saved and stored on the hard disk; however, points and scores are given as feedback on the laptop monitor at the end of each game.
- Type of game: physical, cognitive, action, real-time strategy
- Behavior change procedure used: gamification-based physical-cognitive training enhances extrinsic motivation and engagement in older adults.
- Setting: gamification-based physical-cognitive training can be set in a room environment.
- Device requirements: PC/notebook/laptop with HD webcam
- Sensors used: HD webcam

### Phase IV-V: Implementation and Evaluation Phases

#### Participant Characteristics

In total, 15 community-dwelling older adults aged ≥60 years took part in the study; of the participants, 8 were female and 7 were male. None of the participants had prior experience with game-based training. The demographic characteristics of the participants are presented in Table 2. All participants completed the training program (mean number of exercise sessions 11.07, SD 1.95, with a total of 12 sessions). No falls or other adverse events were reported.

**Table 2.** Characteristics of the study participants.

Characteristics	Mean (SD)	Range (minimum-maximum)
Age (years)	65.27 (4.40)	64.00‐77.00
BMI (kg/m^2^)	24.09 (2.59)	20.03‐28.73
Education level (years)	15.67 (2.58)	9.00‐21.00

#### User Experience in Using the Gamification-Based Exercise System

**Figure 4.**
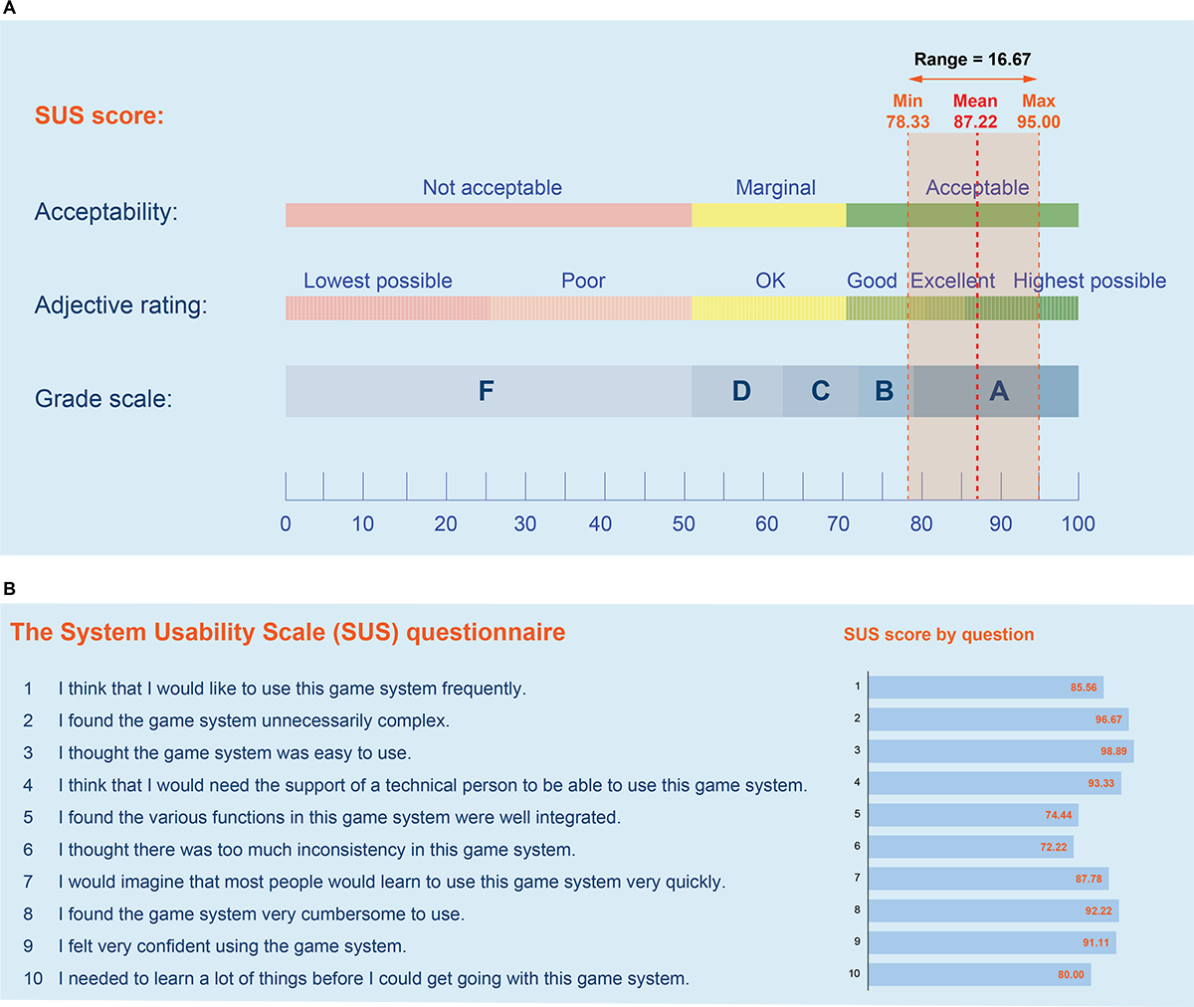
Overview of usability results of the game-based exercise prototype using the SUS questionnaire at the end of the 4-week testing period. (A) The SUS score and its relationship with the grade scale (F, D, C, B, and A), adjective ratings (lowest possible, poor, okay, good, excellent, and highest possible), and acceptability scores (with marginal acceptability at 68.0). (B) The SUS score for each question. SUS: system usability scale.

The user experience in using the game-based exercise was determined using the SUS questionnaire and the PACES. The overall results of the SUS score are illustrated in Figure 4. The mean SUS score was 87.22 (SD 5.76), which corresponds to an A or “excellent to highest possible” acceptance level (based on the grade scale and adjective rating; Figure 4A). Further data exploration showed that, of the 15 participants, 6 rated this gamification-based exercise system prototype as “good,” 5 rated it as “excellent,” and 4 rated it as “highest possible.” The mean SUS score for each question ranged from 72.22 (question item 6) to 98.89 (question item 3), signifying ratings from a marginal “good” to the “highest possible” acceptance level (Figure 4B).

As for the 8-item PACES score, the total PACES score of all items at the end of the first week of training was 44.93 (SD 3.99); the average on each item ranged from 5.27 to 5.93. After 4 weeks, it was 50.53 (SD 4.70); the average on each item ranged from 5.93 to 6.67 (Table 3). A Wilcoxon signed-rank test demonstrated that both the average on each item and total score after the 4-week training were significantly greater than those after 1 week of training (*P*=.001), indicating an improvement in enjoyment and satisfaction after exercise engagement for 4 consecutive weeks.

**Table 3.** The PACES score after 1 week of training and after completion of 4 weeks of training (n=15). All items were rated on a 7-point scale from 1 (strongly disagree) to 7 (strongly agree).

Question items	Week 1, mean (SD)	Week 4, mean (SD)	*P* value^a^
1. I find it pleasurable	5.53 (0.83)	6.33 (0.82)	.003
2. It’s a lot of fun	5.27 (0.88)	5.93 (0.88)	.02
3. It’s very pleasant	5.27 (0.88)	6.27 (0.88)	.002
4. It’s very invigorating	5.60 (0.63)	6.13 (0.83)	.01
5. It’s very gratifying	5.67 (0.62)	6.27 (0.70)	.01
6. It’s very exhilarating	5.73 (0.59)	6.40 (0.74)	.004
7. It’s very stimulating	5.93 (0.59)	6.53 (0.52)	.01
8. It’s very refreshing	5.93 (0.59)	6.67 (0.62)	.005
Total scale of all items (56 points)	44.93 (3.99)	50.53 (4.70)	.001

a Wilcoxon signed-rank test with significant difference at *P*<.05.

#### User Feedback of the Gamification-Based Exercise System

Comments and feedback from the participants on the developed gamification-based exercise system prototype through probing interviews are summarized for each question domain in Table 4.

**Table 4.** The key responses of participants regarding the game characteristics and their perceptions during engagement with the game-based prototype.

Question domains	Positive feedback	Negative feedback
Game mechanics, rules, and interface	The storytelling game (Moving and Memorizing) was enjoyable, with its content, purpose, and display perfectly suited to personal preferences The Ocean Diver game was very enjoyable and well-designed, providing a refreshing experience	It is hard to judge how far to jump to grab animals or objects on time Sometimes in the game, the movements did not align with the actual actions The characters and objects displayed in the game were relatively small Sometimes, the game system froze
Game instructions	Instructions for the game were clear and easy to understand	No negative feedback
Gameplay experience	The exergame was very helpful as it promoted physical activity and memory The exergame could be played at any time, which perfectly fits the schedule There was a noticeable improvement in sleep quality	In the Ocean Diver game, some objects that needed to be collected were placed in tight spots, making it stressful when participants were unable to pick them up
Exercise dosage	The exercise was not too difficult, as it allowed me to keep up with the training	In the first week of training, the legs were quite fatigued; however, after the first week, it went away
Feedback for improvements	Overall, the games were satisfying, enhancing both enjoyment and physical activity	Making the movements in the game better match the user’s actions, increasing the size of the characters and objects, and reducing the game system’s freezing issues

## Discussion

### Principal Findings

This study aimed to design, develop, and determine the user experience of a prototype of low-intensity, gamification-based training targeted at improving physical and cognitive performance in older adults within a home environment using the ADDIE model. The idea of the core concept and platform of the exergame were extracted by integrating the knowledge with well-recognized physical and cognitive outcomes in older adults, considering practical standpoints, and then soliciting critical appraisal by experts. The content validity of the exergames was highly acceptable, demonstrating that the game’s features, rules, and exercise components were well-aligned with both the intended therapeutic goals and practical considerations. The experiences of older adults in terms of usability and enjoyment of the exergame prototype were assessed and their feedback was collected.

The output of this study was the prototype of a gamification-based, interactive physical-cognitive training program in a home-based setting, which consisted of 3 exergames: Ocean Diver, Road Runner, and Moving and Memorizing. Each game had 3 levels of difficulty: beginner, intermediate, and advanced. The user-perceived usability of the game-based exercise prototype was rated as good to excellent, and there were improvements in enjoyment and satisfaction after exercise engagement over 4 consecutive weeks. Additionally, the overall end user feedback indicated that the motivation for engaging in an exergame is its inherent interest and enjoyability.

### Comparison to Prior Work

In recent years, an increasing number of studies have developed and evaluated the effects of exergames on both physical and cognitive functions in older adults. However, the majority of these studies have used moderate-to-high physical intensity levels [19,39,40], which are generally suitable for relatively healthy individuals without significant physical restrictions. Only a limited number of studies focused on developing low-intensity exergames, which may be more practical for older adults who are physically limited or less active, as they require low exertion and low-impact exercise modes. To the best of our knowledge, our game-based exercise is the first prototype of low-intensity, gamification-based, interactive physical-cognitive training specifically designed to enhance both physical and cognitive abilities in older adults. We found that the exergame prototype was usable in a home environment and effectively enhanced the enjoyment and motivation of older adults. The feedback also indicated potential future applications for self-training at home. These findings are in line with previous studies suggesting that computer-based interventions, a delivery platform used in this study, offer a practical, safe, and efficient approach to encouraging adherence to exercise [41,42].

Currently, various approaches to product development may involve end users at different stages of the production process, depending on the specific product requirements and goals. Among various product development approaches, the ADDIE model was selected because it aligns well with our exergame product, which is relatively new and unfamiliar to older adults. This model involves experts in the analysis and design phases (phases I and II) and end users in the implementation and evaluation phases (phases IV and V). Given that experts have knowledge and experience in the fields of physical and cognitive rehabilitation for older adults, as well as game development, they played a key role in the analysis and design phases, which are crucial for achieving both theoretical accuracy and practical feasibility to ensure high-quality outcomes. A representative group of the end users (older adults) tested the exergame prototype and provided feedback during the implementation and evaluation phases. The feedback and suggestions from end users were essential in shaping the final product, guaranteeing that the prototype was closely tailored to their needs and preferences. A previous study suggested that individualization of the exercise in terms of needs and interests is necessary to enhance individual engagement, thereby contributing to positive intervention outcomes [43]. In addition, this study utilized a computer vision–based system as a technology platform to deliver an exercise program (in the form of an exergame) in a home-based environment, aiming to provide accessibility and ease of integration into users’ lives. Previous findings have suggested that home-based exercise exerts greater exercise engagement than center-based exercise due to its accessibility and flexibility in the schedule, allowing individuals to integrate it conveniently into their daily life [44,45]. Taken together, the benefits of using the ADDIE model approach and delivering the platform as a computer vision–based system for the development of an exergame prototype may represent a potential strategy for enhancing the enjoyment, motivation, and engagement of older adults in exercise in a home-based setting.

Regarding the user experience with the game-based training system, participants reported increased enjoyment, as indicated by their PACES scores, and provided positive feedback on the enhancement of both their enjoyment and physical activity. The increase in the enjoyment level of participants may, at least in part, be attributed to several fundamental aspects of the game. These include game elements that provide real-time feedback (ie, scores and rewards), game mechanics (ie, real-time interface and sound effects), and game rules (optimal difficulty level, grading from simple to advanced), which in turn results in high enjoyment and self-efficacy empowerment. Our findings are supported by prior research, which suggests that intrasession feedback, positive reward, and the use of graded exercise may enhance intrinsic motivation and self-confidence in the capability to exercise, potentially reinforcing repetitive desirable behavior [46,47]. Some negative feedback on the game’s characteristics was received, including issues with asynchronization between user actions and the display, system freezing, and the need to enlarge characters and objects. Thus, additional adjustments to the game mechanics are necessary prior to its deployment for end users. Apart from the game characteristics, the usability of a game-based system is a key aspect in enhancing acceptability and exercise adherence. As we found, the users’ perception of the usability of the developed game-based training system, as determined by the SUS score, ranged from good to highest possible. The positive response from usability testing may potentially be due to the game interface and operations being specifically designed to be used independently by older adults. Consistently, a previous study has suggested that the feature design of exergames, particularly an interface that is friendly to older users, is a crucial factor contributing to the positive acceptance of older users who are unfamiliar with new technology [48]. Nonetheless, the lowest scores on the SUS questionnaire were found in items 5 and 6 (scoring 74.44 and 72.22, respectively), which are related to game mechanics and consistent with feedback for improvements. Therefore, further refinement of the exergame system is warranted before implementation for end users, particularly concerning technical errors and game mechanics issues encountered by participants.

### Strengths and Limitations

To the best of our knowledge, this game-based home exercise is the first prototype customized for older adults with low physical capacity, aiming to simultaneously stimulate specific cognitive domains and physical components. Despite the positive results, this study has certain limitations that need to be addressed. First, participants in this study had relatively high educational backgrounds and were socially active, which may introduce potential bias, as they may be more familiar with technology. Further, they did not exhibit obvious physical restrictions as the study inclusion/exclusion criteria were set to minimize potential adverse events during participation. Second, the sample size was relatively small. Together, the findings may not be generalizable to a broader older adult population and should be considered preliminary, warranting cautious interpretation. Third, exercise adherence was observed over a 4-week training period; hence, long-term adherence remains unknown. Last, since this study focused on developing and assessing the usability of an exergame prototype for home-based settings, hard outcomes, such as physical and cognitive parameters in response to exergame engagement, have not yet been established. Further research that includes larger sample sizes, diverse demographic backgrounds, extended training periods, and examination of effectiveness through comprehensive outcomes is needed.

### Future Directions

The outcome of this study is the prototype of a home-based, low-intensity exercise program that is practical and has the potential to enhance the enjoyment and motivation of older adults. The next step will be to refine the game-based prototype based on end user critical feedback and examine its effectiveness in promoting the physical and cognitive functions of community-dwelling older adults, thereby giving this new type of exergame a promising future.

### Conclusions

In this study, a low-intensity, gamification-based, interactive physical-cognitive training system was developed for older adults with limited capacity to engage in moderate-to-high intensity exercise. The exergame prototype, delivered via a computer vision–based platform for a home-based, self-training exercise, was well-received by the end users for its usability and enjoyment. Although these findings hold promise for implementing the exergame in the target population, further research is warranted to determine its effectiveness in promoting physical and cognitive functions.

## Supplementary material

10.2196/59141 Multimedia Appendix 1 Content validity assessment form for experts.

10.2196/59141 Multimedia Appendix 2 Landmark of BlazePose 2D/3D pose estimation.

10.2196/59141 Multimedia Appendix 3 Short Physical Performance Battery scoring sheet.

10.2196/59141 Multimedia Appendix 4 Environmental configuration of the gamification-based interactive physical-cognitive training system.

10.2196/59141 Multimedia Appendix 5 The probing questions on perceptions of the game characteristics and user experiences during engagement with the game-based training system prototype.

## Abbreviations

ADDIE: analysis, design, development, implementation, and evaluation
HD: high definition
IOC: index of item-objective congruence
PACES: Physical Activity Enjoyment Scale
SUS: system usability scale
