# Supplementary material for: Home-Based, Low-Intensity, Gamification-Based, Interactive Physical-Cognitive Training for Older Adults Using the ADDIE Model: Design, Development, and Evaluation of User Experience
Source: JMIR Serious Games. 2024 Oct 29;12:e59141. doi: 10.2196/59141 (PMC11536494; doi:10.2196/59141)
Supplement: Multimedia Appendix 1 [file games-v12-e59141-s001.pdf]

# Content validity assessment form for experts

**Please assess the consistency and appropriateness of each item or component of the gamification-based interactive, low-intensity, combined physical-cognitive exercise. Provide your opinion using the rating scale below and suggest improvements in the comments section.**
**The scoring criteria are as follows: +1 = consistent/appropriate, 0 = not sure, and -1 = inconsistent/inappropriate**

## 1. Is the game consistent with its objectives?

| Game | Training Purpose | Training Description | Rating | | | Comments |
|---|---|---|---|---|---|---|
| | | | +1 | 0 | -1 | |
| **Game 1: Ocean Diver**<br><br>Duration: 12 min | 1.1 To improve memory and executive function, including planning, sequencing, inhibiting, and planning ability, as well as attention. | Swim to collect the list of pre-determined objects in the correct order | | | | |
| | 1.2 To improve physical activity | Swimming is controlled by moving UE (like frog stroke style). Moving to the side is controlled by stepping to the left or right. | | | | |
| **Game 2: Road Runner**<br><br>Duration: 12 min | 2.1 To improve memory and visuospatial ability | 2.1 Running to collect predetermined objects as many as possible (no order) | | | | |
| | 2.2 To improve physical activity | 2.2 Running is controlled by moving UE (as during running). Moving to the side is done by stepping to the left or right. Collecting the object is done by raising either the left or right arm up. | | | | |

| Game | Training Purpose | Training Description | Rating | | | Comments |
|---|---|---|---|---|---|---|
| | | | **+1** | **0** | **-1** | |
| **Game 3: Moving & Memorizing**<br><br>Duration: 12 min | 3.1 To improve memory (delayed recall) and attention. | 3.1 Listen to a story while walking to collect coins. Then answer questions related to the story at the end of the game. | | | | |
| | 3.2 To improve physical activity | 3.2 Stepping to the left or right to collect coins. | | | | |
| **Note:** The game system features various categories of objects (e.g., vegetables, fruits, animals, kitchenware, clothing items etc.) with each category containing multiple types. The objects that appear will be randomly drawn from these categories. | | | | | | |

## 2. Is the difficulty level of the game suitable for older adults?

| Game | Training Description | Rating | | | Comments |
|---|---|---|---|---|---|
| | | +1 | 0 | -1 | |
| **Game 1: Ocean Diver** | 2.1 Difficulty level of game 1:<br>Game Difficulty Levels<br>Level 1: Collect designated items in the correct and complete order (there will be some obstacles in which the player must navigate and avoid).<br>Level 2: Increases the difficulty from Level 1 by having other unrelated items floating in the water. If they pick them incorrectly, 1 point will be deducted each time.<br>Level 3: Increases the difficulty from Level 2 by having the items required to be collected in the correct and complete order swapped with coins. (Items-Coins-Items-Coins, etc.)<br>Note: Players must score 50 percent to unlock the next level. | | | | |
| **Game 2: Road runner** | 2.2 Difficulty level of game 2:<br>Level 1: Collect 5 types of predetermined fruit from other irrelevant items (disruptors). If they collect the wrong item, 1 point will be deducted. | | | | |

| Game | Training Description | Rating | | | Comments |
|---|---|---|---|---|---|
| | | +1 | 0 | -1 | |
| | Level 2: Collect 5 types of predetermined animals from other irrelevant items (disruptors). Additionally, there will be obstacles along the road in which the player must navigate and avoid. If they collect the wrong item, 1 point will be deducted.<br>Level 3: Collect 5 mixed types of predetermined animals and fruit from other irrelevant items (disruptors). There will also be obstacles along the road in which the player must navigate and avoid. If they collect the wrong item, 1 point will be deducted.<br>Note: Players must score 50 percent to unlock the next level. | | | | |
| **Game 3: Moving and memorizing** | 2.3 Difficulty level of game 3:<br>There are three levels of difficulty based on the story being listened to. The story will be increasingly complicated with uncommon/ unfamiliar vocabularies.<br>Note: Players must answer at least 3 out of 4 questions correctly to unlock the next level. | | | | |

## 3. Overall, is the game suitable for older adults, considering safety, principles of exercise, physical and cognition benefits, etc?

| Items | Rating | | | Comments |
|---|---|---|---|---|
| | **+1** | **0** | **-1** | |
| Overview of Brain-Body Fit (BB-Fit) | | | | |

## 4. Other suggestions (if any)

…………………………………………………………………………………………………………………………

…………………………………………………………………………………………………………………………

…………………………………………………………………………………………………………………………
