# Supplementary figures and images for: Home-Based, Low-Intensity, Gamification-Based, Interactive Physical-Cognitive Training for Older Adults Using the ADDIE Model: Design, Development, and Evaluation of User Experience
Source: JMIR Serious Games. 2024 Oct 29;12:e59141. doi: 10.2196/59141 (PMC11536494; doi:10.2196/59141)

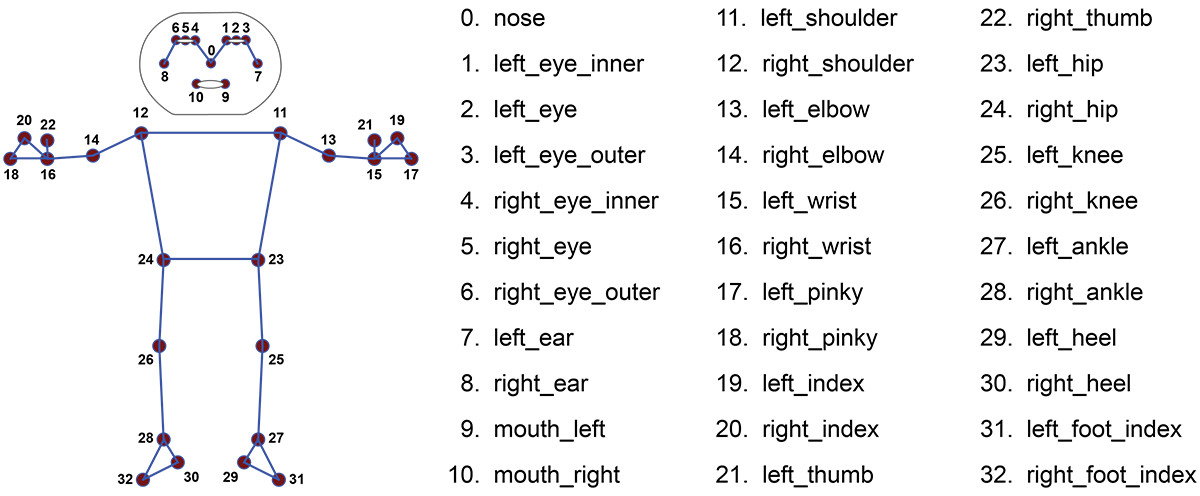

Supplement: Multimedia Appendix 2 [file games-v12-e59141-s002.png]

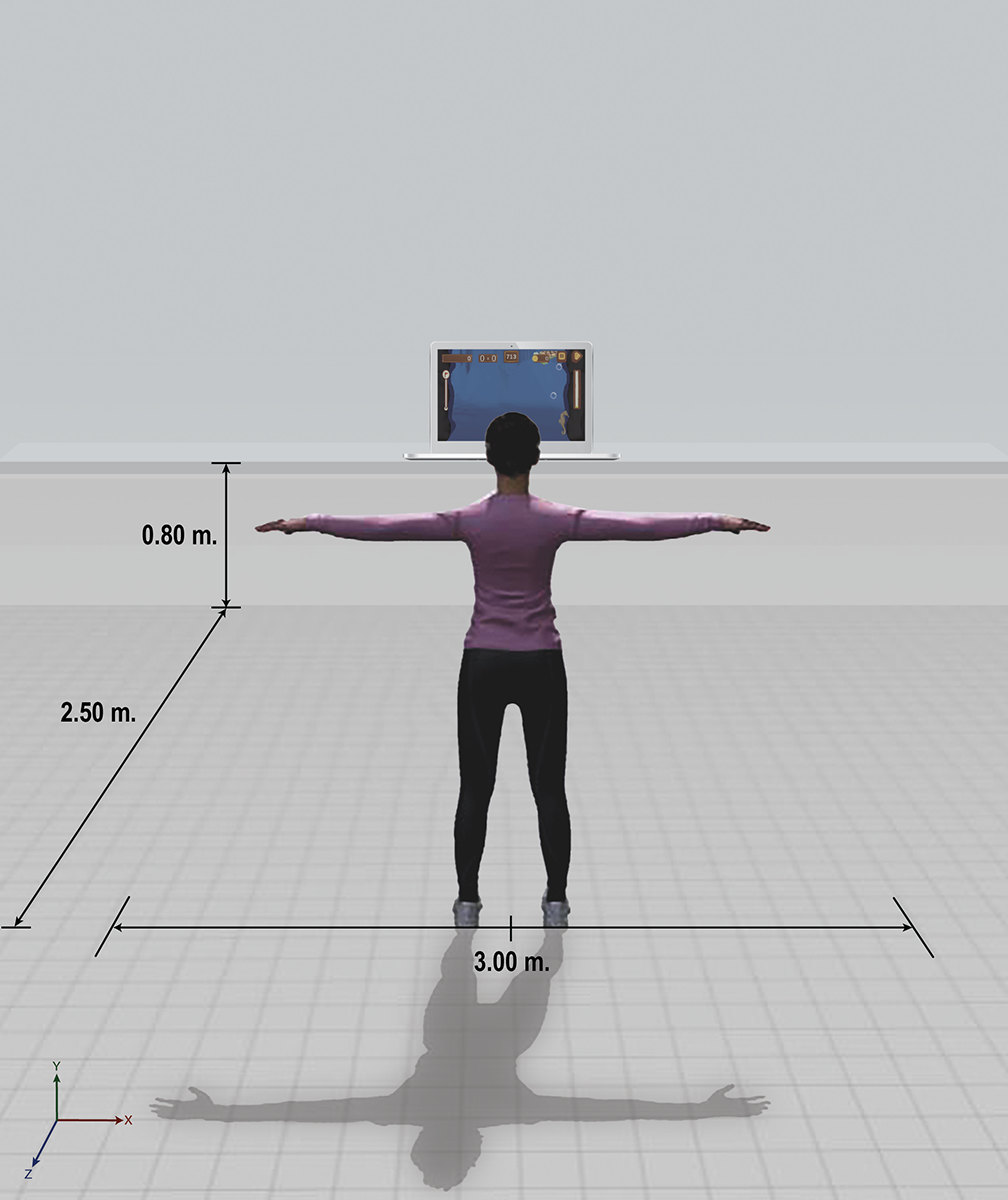

Supplement: Multimedia Appendix 4 [file games-v12-e59141-s004.png]
