# Supplementary material for: Home-Based, Low-Intensity, Gamification-Based, Interactive Physical-Cognitive Training for Older Adults Using the ADDIE Model: Design, Development, and Evaluation of User Experience
Source: JMIR Serious Games. 2024 Oct 29;12:e59141. doi: 10.2196/59141 (PMC11536494; doi:10.2196/59141)
Supplement: Multimedia Appendix 3 [file games-v12-e59141-s003.pdf]

Short Physical Performance Battery (SPPB) - scoring sheet

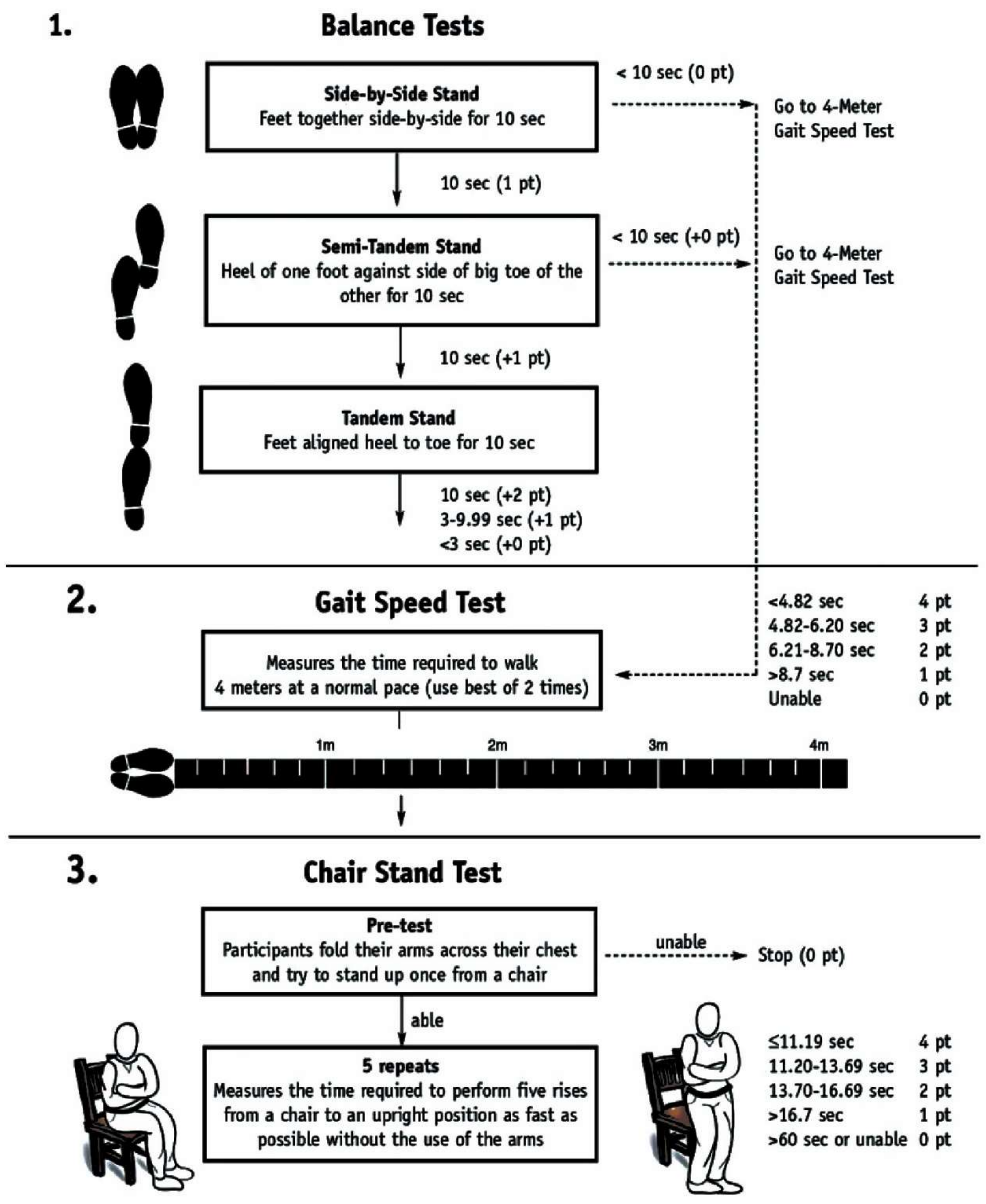

Source: Wall chart courtesy of Dr. Jack Guralnik.

Test	Score
1. Balance Tests	
2. Gait Speed Test	
3. Chair Stand Test (5STS)	
**Total**	
