# Supplementary material for: Home-Based, Low-Intensity, Gamification-Based, Interactive Physical-Cognitive Training for Older Adults Using the ADDIE Model: Design, Development, and Evaluation of User Experience
Source: JMIR Serious Games. 2024 Oct 29;12:e59141. doi: 10.2196/59141 (PMC11536494; doi:10.2196/59141)
Supplement: Multimedia Appendix 5 [file games-v12-e59141-s005.pdf]

Probe questions on perceptions of the game characteristics and user experiences during engagement with the exergame prototype

Question domains	Question interview
Game mechanics, rules, and interface	• "Were there any elements of the interface that you found particularly helpful or problematic?"<br>• "Were there any features or controls that were confusing or difficult to use?"<br>• "Did you encounter any difficulties in following them?"
Game instructions	• "Were the rules and instructions for the game clear and easy to understand?"
Gameplay experience	• "How did you find the overall experience of playing the exergame?"<br>• "Did the exergame keep you engaged and motivated throughout the session?<br>• "Were there any aspects that made you feel uncomfortable, such as stress or adverse effects like muscle fatigue?"
Exercise dosage	• "Was the level of exercise too hard?"<br>• "Did you find the exercise dosage appropriate for individuals who have restrictions on engaging in high-to-moderate intensity exercises, such as physical limitations, comorbidities, or inactive aging?"
Feedback for improvements	• "Do you have any suggestions for improving the game-based prototype?"<br>• "Are there any features or elements you think should be added or changed before implementing it with the end-users?"
